# Supplementary material for: Modulating Activity Evaluation of Gut Microbiota with Versatile Toluquinol
Source: Int J Mol Sci. 2022 Sep 14;23(18):10700. doi: 10.3390/ijms231810700 (PMC9505934; doi:10.3390/ijms231810700)
Supplement: Supplementary file 1 [file ijms-23-10700-s001.zip › ijms-1905794-supplementary.pdf]

Supporting Information

Modulating Activity Evaluation of Gut Microbiota with Versatile Toluquinol

Long-Long Zhang,[†] Ya-Jun Liu,[†] Yong-Hong Chen, Zhuang Wu, Bo-Ran Liu, Qian-Yi Cheng, Ke-Qin Zhang, Xue-Mei Niu*

State Key Laboratory for Conservation and Utilization of Bio-Resources & Key Laboratory for Microbial Resources of the Ministry of Education, School of life Sciences, Yunnan University, Kunming, 650091, People's Republic of China

[†]These authors contributed equally to this work

Corresponding Author Xue-Mei Niu: Phone: 86- 871-65032538; Fax: 86-871- 65034838;

Email: xmniu@ynu.edu.cn; orcid.org/0000-0002-8977-8956

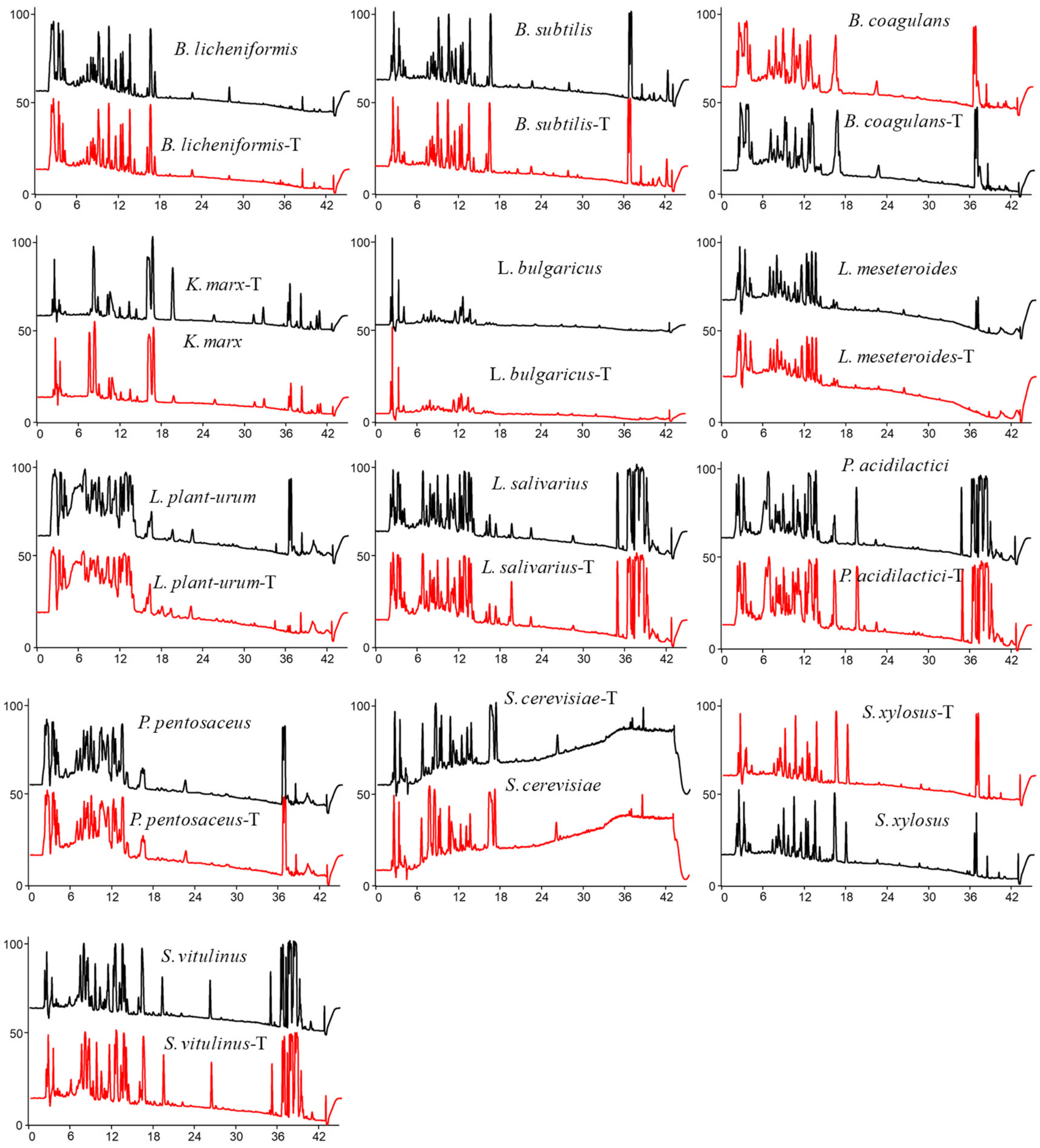

Figure S1. Comparison of metabolic profiles of the 13 gut microbes fed with and without toluquinol suggested no transformation of toluquinol in 13 strains including *Bacillus coagulans Bacillus licheniformis*, *Bacillus subtilis*, *Kluyveromyces marx*, *Leuconstoc meseteroides*, *Pediococcus acidilactici*, *Saccharomyces cerevisiae*, *Lactobacillus bulgaricus*, *Lactococcus lactis subsp. Lactis*, *Lactobacillus planturum*, *Lactobacillus salivarius*, *Pediococcus pentosaceus*, *Staphylococcus vitulinus*, *Staphylococcus xylosus*, and S*tretpococcus thermophilus*.

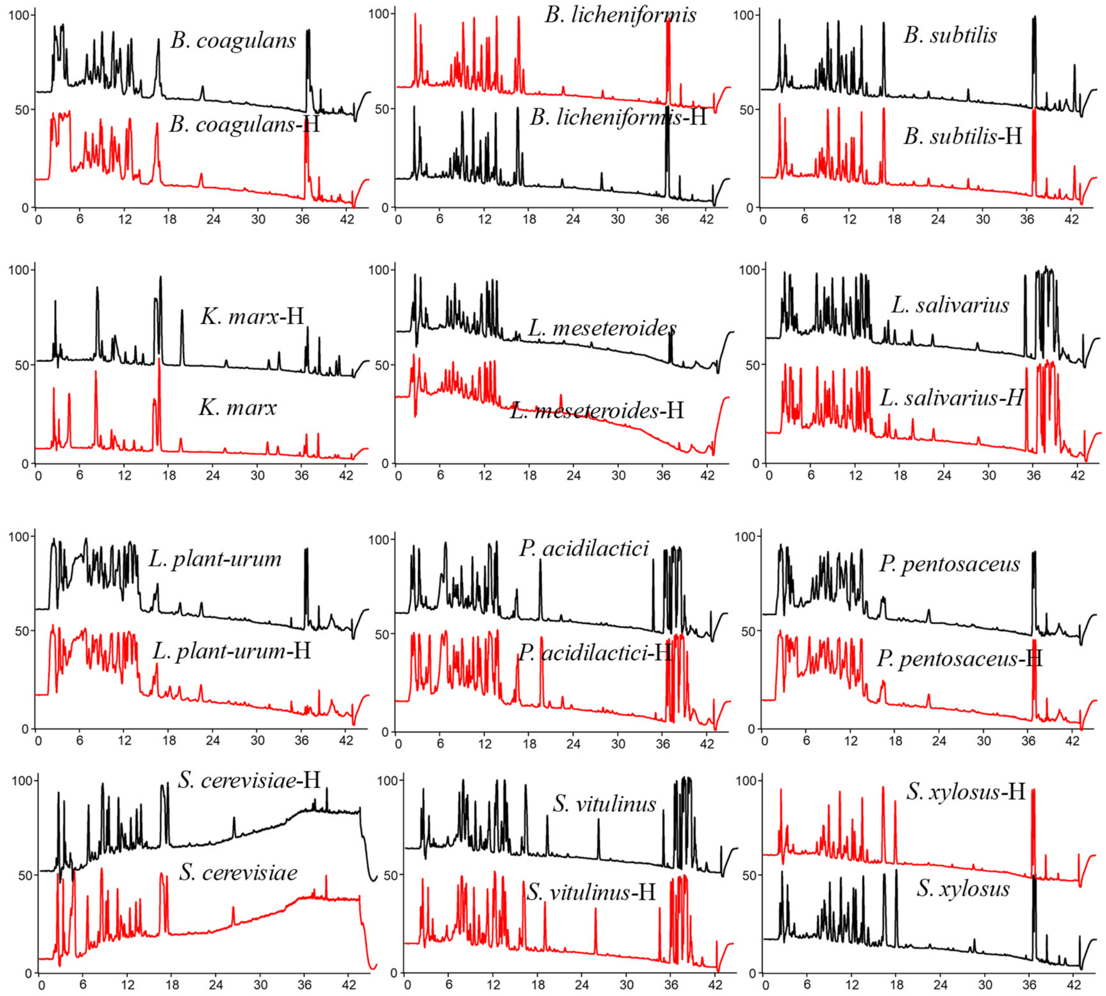

Figure S2. Comparison of metabolic profiles of 12 gut microbes fed with and without hydroquinone (H) suggested no transformation of hydroquinone in these 12 strains including *Bacillus coagulans Bacillus licheniformis*, *Bacillus subtilis*, *Kluyveromyces marx*, *Leuconstoc meseteroides*, *Pediococcus acidilactici*, *Saccharomyces cerevisiae*, *Lactobacillus bulgaricus*, *Lactococcus lactis subsp. Lactis*, *Lactobacillus planturum*, *Lactobacillus salivarius* , *Pediococcus pentosaceus*, *Staphylococcus vitulinus*, and *Staphylococcus xylosus*.

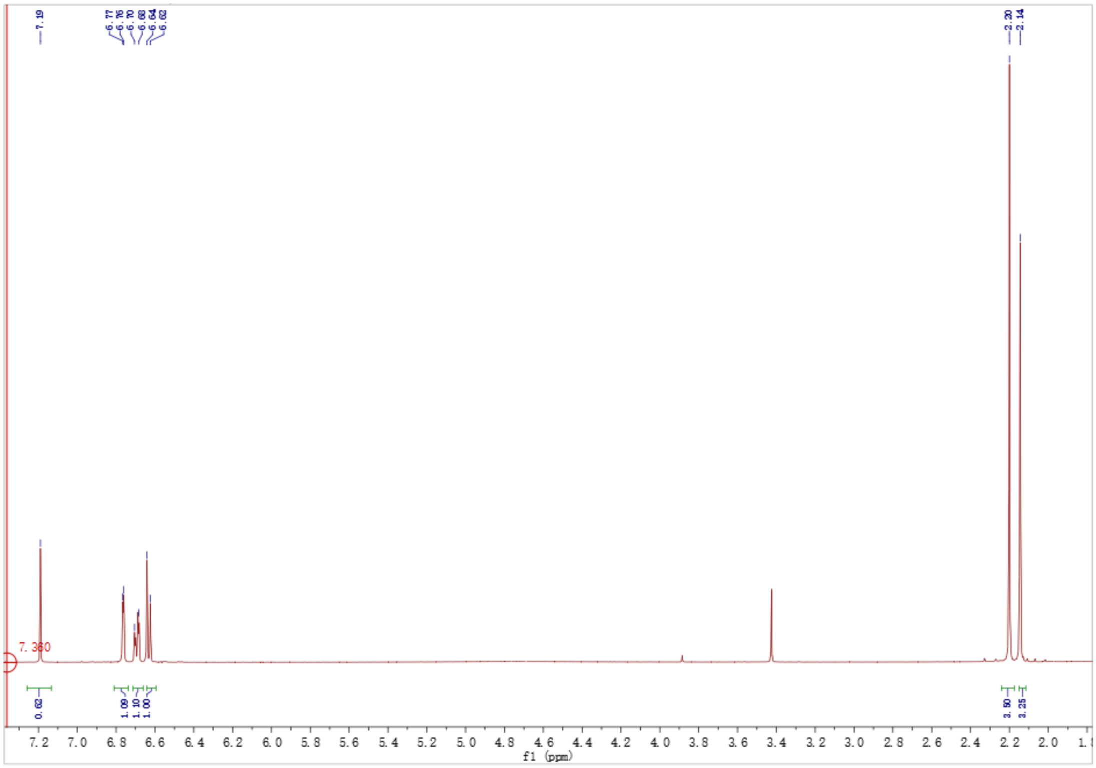

Figure S3. The ^{1}H NMR (500 MHz) spectrum of compound 1 recorded in $CDCl_3$.

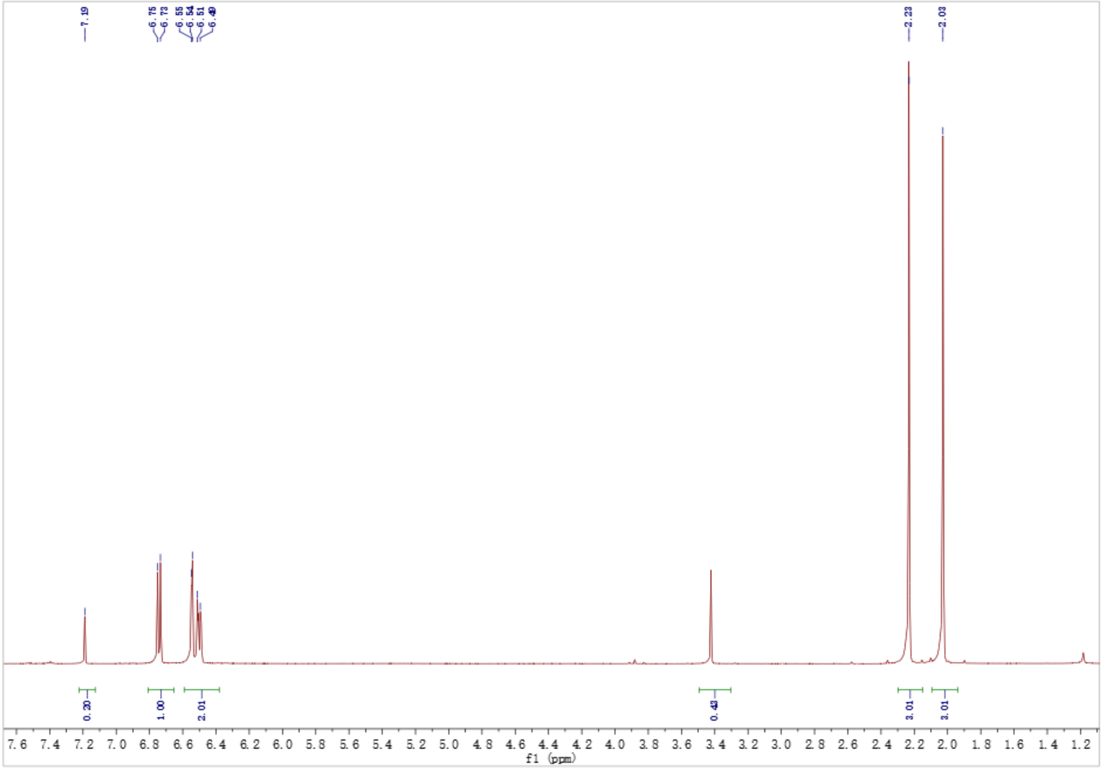

Figure S4. The ^{1}H NMR (500 MHz) spectrum of compound 2 recorded in $CDCl_3$.

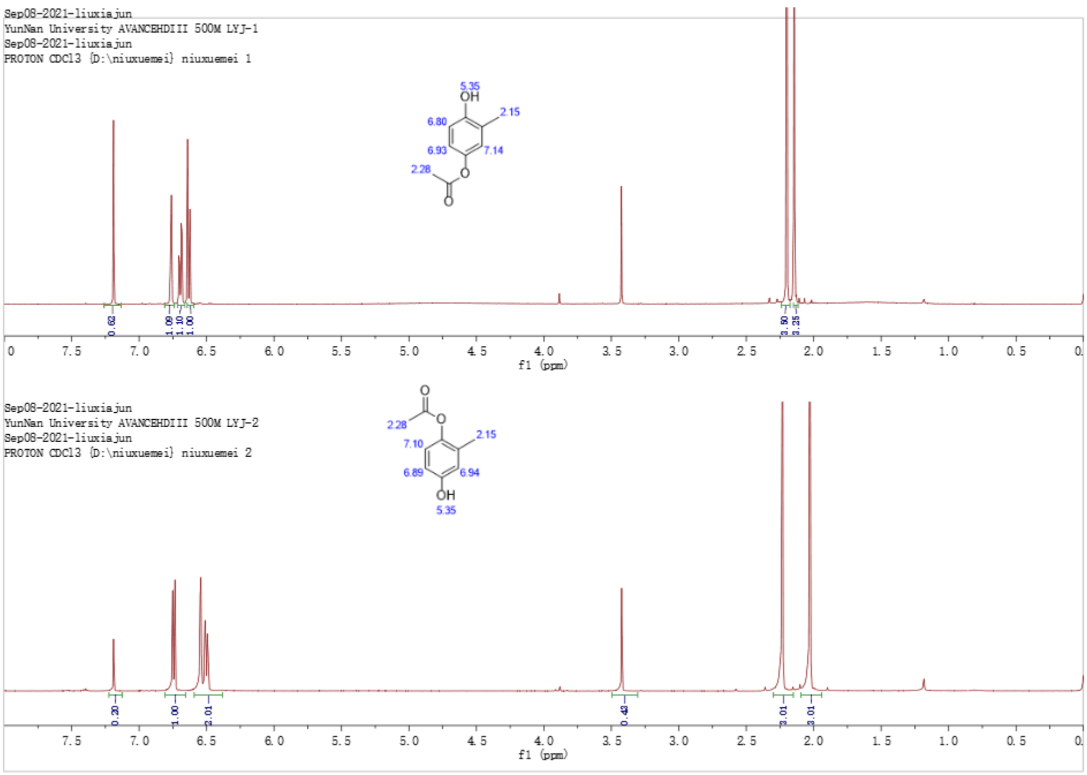

Figure S5. Comparison of the ^{1}H NMR (500 MHz) spectra of compounds 1-2 recorded in $CDCl_3$.

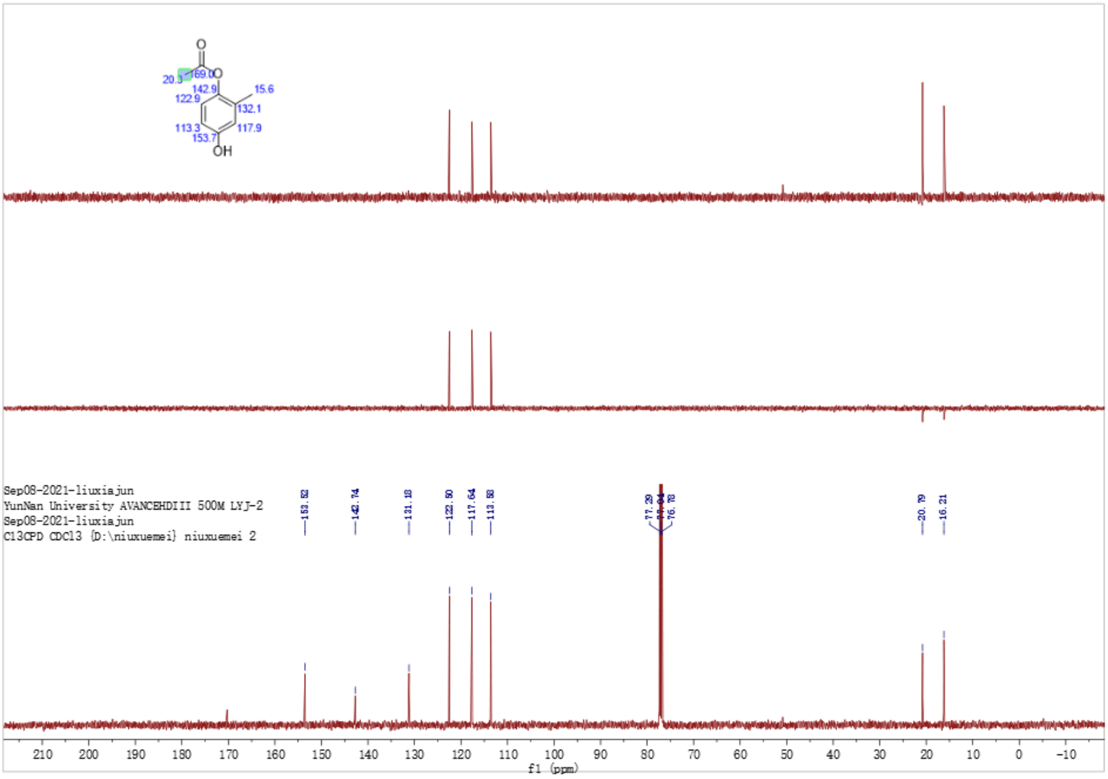

Figure S6. The ^{13}C NMR (125 MHz) spectrum of compound 2 recorded in $CDCl_3$.

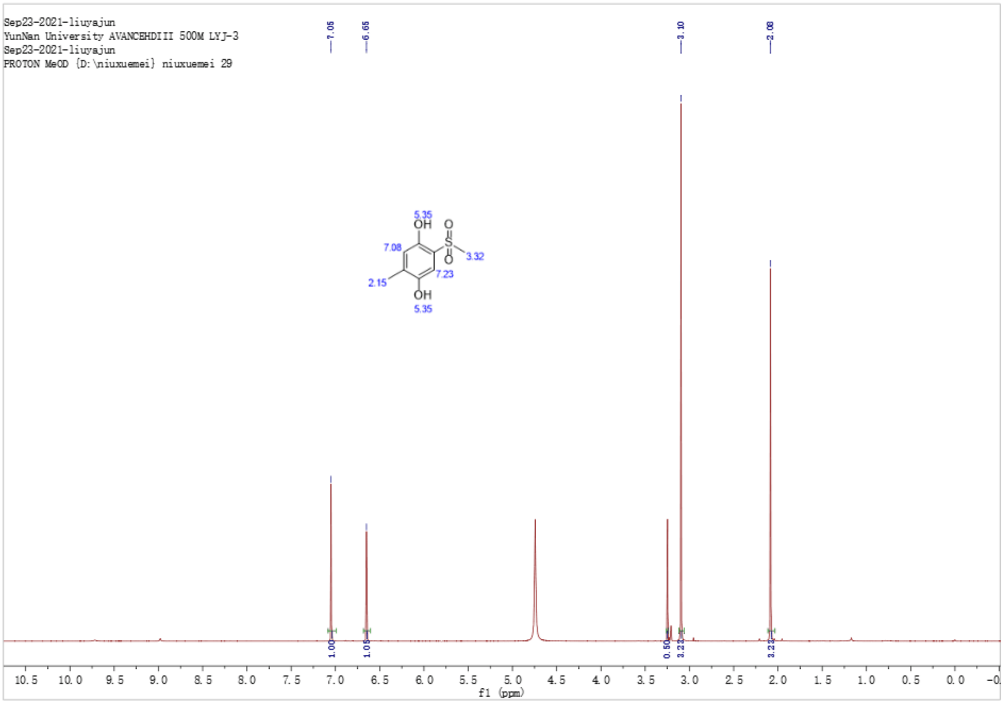

Figure S7. The ^{1}H NMR (500 MHz) spectrum of compound 3 recorded in MeOD.

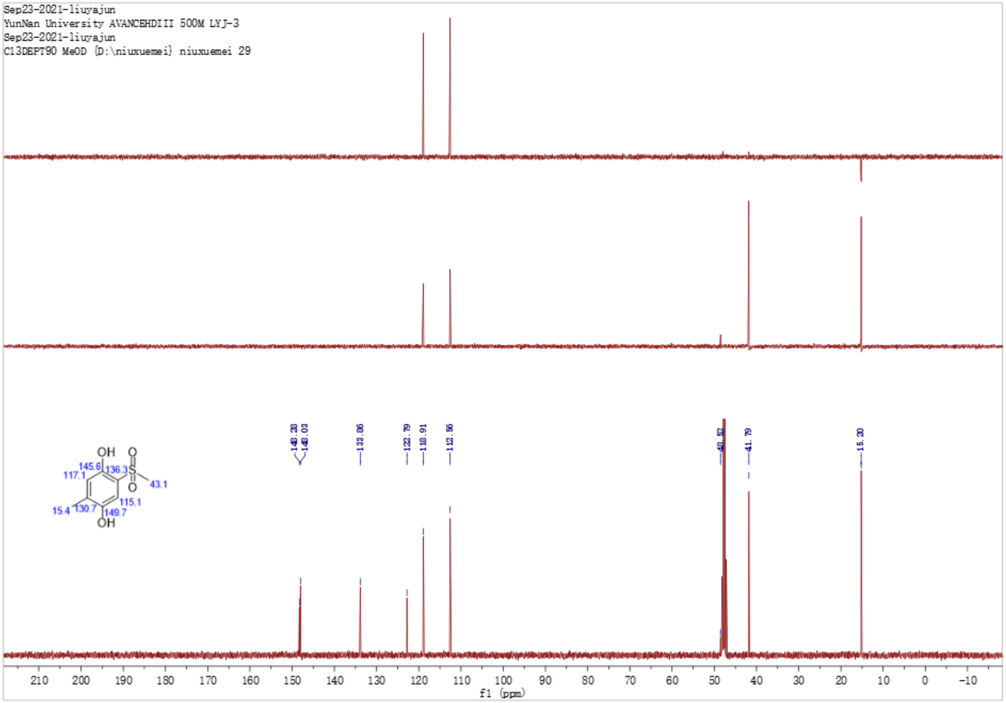

Figure S8. The ^{13}C NMR (125 MHz) spectrum of compound 3 recorded in MeOD.
